# Effects of supportive counseling using a positive psychology approach on coping patterns among pregnant women with nausea and vomiting

**DOI:** 10.1186/s12884-022-04603-4

**Published:** 2022-03-27

**Authors:** Mina Abbasi, Azam Maleki, Loghman Ebrahimi, Behnaz Molaei

**Affiliations:** 1 Zanjan University of Medical Sciences, Zanjan, Iran; 2 Social Determinants of Health Research Center, Zanjan University of Medical Sciences, Azadi Square, Jomhori Eslami St, 4515613191 Zanjan, Iran; 3 Department of Psychology, Faculty of Humanities, University of Zanjan, Zanjan, Iran; 4 Fellowship of Perinatology, Obstetrics & Gynecology, Zanjan University of Medical Sciences, Zanjan, Iran

**Keywords:** Positive psychology, Compatibility, Pregnancy nausea and vomiting, Women's health

## Abstract

**Background:**

Pregnancy nausea and vomiting (NVP) are associated with a wide range of physical and mental changes in the mothers and could affect their adaptation to pregnancy. There is a gap of knowledge regarding the effectiveness of a positive psychology approach counselling on improving coping patterns in women with NVP.

**Objective:**

This study aimed to determine the effect of supportive counselling with a positive psychology approach on improving coping patterns in women with NVP.

**Method:**

The current study was a randomized controlled trial on 60 pregnant women who experienced nausea and vomiting in Zanjan a province in the northwest of Iran, 2020–2021. Using four-block random sampling, eligible women were randomly assigned to the intervention and control groups. According to the Seligman protocol, the intervention group received supportive counselling in six sessions of 45 min3 times a week. The revised prenatal coping inventory (Nu PCI) was used to collect data and analyzed using Chi-square, repeated measures ANOVA, and MANOVA at a 95% confidence level.

**Results:**

In the counseling group, the mean (SD) of a total score of coping with pregnancy before the intervention was 6.11 (1.31) which increased to 8.40 (1.03) in 4 weeks after the intervention. Based on the MANOVA test with adjusting the number of pregnancies, the mean total score of coping with pregnancy was statistically significant 4 weeks after the intervention compared with the control group (Adjusted mean difference (AMD) = -1.84, CI95% -2.36, -1.32, *p* = 0.001).

**Conclusion:**

The use of positive psychology in supportive counselling improved the coping of mothers suffering from nausea and vomiting during pregnancy. It is recommended that this approach be incorporated into prenatal care to promote the health of pregnant women.

**Trial registration:**

The study was registered at the Iranian Registry of Clinical Trials website under the code IRCT20150731023423N17.

Registration date: 2020–10-31, Expected recruitment start date: 2020–11-01.

IRCT Id: IRCT20150731023423N17.

**Supplementary Information:**

The online version contains supplementary material available at 10.1186/s12884-022-04603-4.

## Introduction

Pregnancy nausea and vomiting (NVP) is a common complaint in the first half of pregnancy, affecting 50–90% of pregnant women to varying degrees. Nausea, vomiting, gagging, dizziness, increased sensitivity to odour, increased cravings for certain foods, changes in daily functioning, mood, and fatigue are among the symptoms [1]. The cause of NVP is unknown and multifactorial in which biological, psychological, social, economic, and psychological factors can all be involved [2].

Lack of timely treatment of NVP results in severe maternal and fetal complications, such as low birth weight, preterm delivery, and reduces the quality of life in women [3]. Additionally, hyperemesis gravidarum and low quality of life negatively affect coping with pregnancy and the role of motherhood [4]. A poorer adaptation to pregnancy is associated with prenatal stress, depression, and anxiety [5]. On the other hand, poor psychological adjustment was directly related to the severity of nausea and vomiting during pregnancy and the perceived stress level was inversely related to the perceived social support [6].

Pregnancy adaptation refers to selecting and implementing coping strategies appropriate to the stressors encountered during pregnancy [6, 7]. Isbir et al. (2013) reported that women use a variety of adaptation patterns to cope with nausea and vomiting in pregnancy, including changes in self-concept, changes in living function, and changes in emotional interdependence with spouse and others [8]. Treatment for pregnancy nausea and vomiting includes lifestyle modifications, medication, supportive therapies, and psychological interventions such as hypnosis, behavioral therapy, and cognitive-behavioral therapy with mindfulness (CBTM), which all have varying degrees of effectiveness in controlling the symptoms [9].

Pregnant mothers' concern about the side effects of drug therapy on fetal health has led to women opting for non-pharmacological methods to alleviate nausea and vomiting symptoms [10]. Today, as the emphasis shifts from disease treatment to prevention and health promotion, modern theories such as positive psychology are gaining traction/support [11]. Martin Seligman introduced the positive psychology approach in 2000, where he emphasized positive traits, psychological capital, and the ability to better adapt to and cope with life's events. This method is comparable to other treatments such as pharmaceutical treatment [12]. To deal with pregnancy challenges such as pregnancy-related nausea and vomiting requires some kind of physical and mental adaptation [5]. There are several reasons why this method is effective in reducing negative emotions but not coping with pregnancy among women with NVP. By thinking positively, we perceive the stress as less threatening, can cope with it effectively [13]. The training exercises in this approach have a direct influence on interpersonal relationships, self-awareness of abilities and strengths, which increases self-confidence and reduces stress and anxiety [12]. However, there is a gap of knowledge regarding the effectiveness of this approach as a supportive method in the pattern of coping with various aspects of pregnancy and childbirth, particularly in improving the adaptation patterns of women with NVP. The present study was conducted aimed to determine the effect of supportive counselling with a positive psychology approach on improving coping patterns in pregnant women who experienced nausea and vomiting.

## Method

### Design of study and setting

The present study was a controlled randomized controlled trial that was conducted from October 2020 to May 2021. The research setting was the comprehensive health centers of Zanjan a city in the northwest of Iran. Zanjan city has 35 health centers. Ten health centers were selected randomly. The research population included pregnant women with NVP who registered in the mentioned health centers. For enrolment of eligible women, the phone number of pregnant women obtained through a national electronic identification system (CIB), if there are no eligible people, or did not want to participate in the study, or did not answer the phone number after two calls the selected number is removed and the next number replaced.

### Participants

Inclusion criteria included a Rhode’s score of mild to moderate nausea and vomiting severity, gestational age of 6–10 weeks, the chronological age of 18–35 years, Literate, having a contact phone number, and a wanted pregnancy; the exclusion criteria before random assignment of groups comprised pregnancy complications, gastrointestinal disease, the experience of traumatic, and stressful events during the last 3 months.

We do not access a similar study showing the effectiveness of positive psychology counseling on coping with pregnancy among women with NVP. The sample size was calculated at 4 people based on the study conducted by Mostafaei et al. in 2019 [14]. The calculated sample size was very small. Therefore, the sample size was calculated by a pilot study (9 people in the intervention group, 11 people in the control group). With considering the 95% confidence level (Z1-α = 1.96), the test power of 80% (Z1-β = 0.85) and based on coping variable with the mean and standard deviation in the intervention group (M_1_ = 4.87and S_1_ = 2.69) and control (M_2_ = 3.11 and S_2_ = 1.96) and using the formula for calculating the sample size in two independent groups, the total sample size was calculated to be 27. Taking into account the 10% drop in the sample, the final sample size was 30 participants in each group.

### Sampling method

**Fig. 1:**
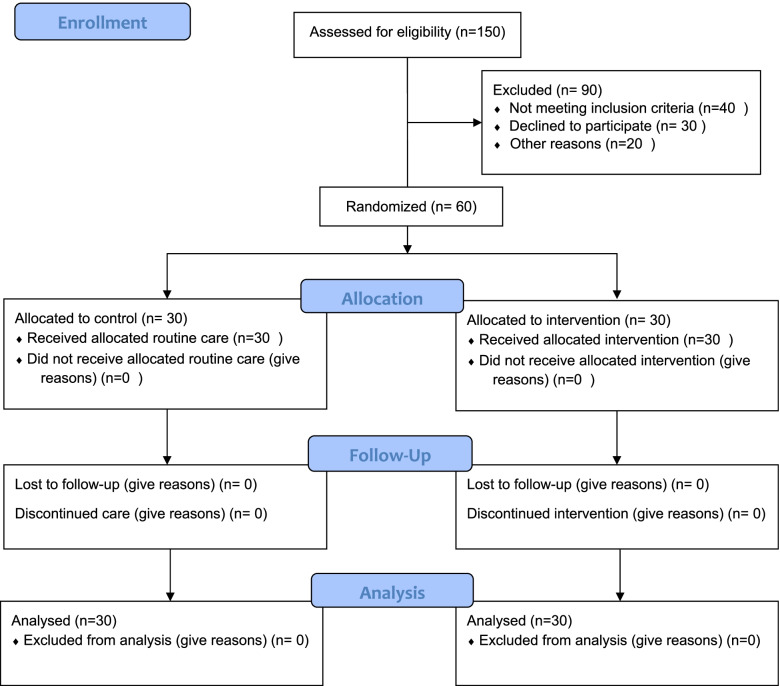
The process of participants enrolment

Among 150 pregnant women evaluated by the researcher, sixty women met the eligible criteria, and ninety people have excluded from the study. All participants were allocated to intervention and control groups using a randomized four-block method. For this reason, two allocations to the intervention group (AA) and two allocations to the control group (BB) were considered in four blocks, resulting in a total of six modes. The sample size was chosen from the created four-block table of random numbers to reach 60 persons. The created random sequences were recorded on the card, and the cards were placed in the letter envelopes in the correct order. To establish a random sequence, the envelopes were all numbered the same way on the outside. Finally, the letter envelope lids were glued together and arranged in a box, respectively. As soon as the eligible participants entered the study, one of the envelopes was opened in order, revealing the participant's allocated group (See Fig. 1 the process of sampling presented).

### Intervention

The intervention group received supportive counselling individually in 6 sessions (45–60 min each), based on the positive psychology protocol developed by Tayyab Rashid and Martin Seligman [15]. Meetings were held three times a week. The counseling sessions were conducted by the first author who had passed a course on positive psychology counseling in a private clinic in Tehran. Then, a positive psychology content program was developed by the researchers. Counselling was held individually in a place with adequate space to observe social distance and other health protocols to prevent Covid-19 diseases such as proper air conditioning, disinfection surfaces with alcohol, and face mask. There was no attrition in the study and after the interventions. Table 1 shows the content of the consultation sessions separately.

**Table 1:** Positive psychological counselling treatment protocol to improve pregnancy adaptation patterns

**Session**	**Session topic**	**Session content**	**Session homework**
First	an introduction to positive psychology and familiarity with 24 capabilities	• Introduction to Positive Psychology, knowledge of 24 strengths or capabilities, explanation of its goals • Teaching about the physiological changes that occur during pregnancy, such as nausea and vomiting • Midwifery care training to reduce physical and mental annoyances per health protocol, such as nausea and vomiting during pregnancy	Name a few of your abilities and explain them using the relevant worksheet
Second	Introducing the three paths leading to happiness	• A study of mothers' attitudes toward pregnancy and negative pregnancy beliefs • Teaching enjoyable pregnancy imagery • Describing the causes of pregnancy nausea and vomiting • Describing the three paths to happiness (pleasure, commitment, and meaning) and 4 sources of enjoyment for reducing stress and increasing adaptability	1. Positive self-introduction 2. Completion of an active values questionnaire in practice www.viacharacter.org
Third	Positive emotions and enjoyment	• A comprehensive description of positive and negative emotions, and their effects on people's lives • Improving mothers' mental and physical abilities to help them adjust to pregnancy • Explanations of the role of positive emotions in improving well-being and life quality, along with neutralizing negative emotions, maturation, and prosperity	Improving mothers' mental and physical abilities to improve pregnancy outcomes exercise-related nausea and vomiting
Fourth	Forgiveness and gratitude	• Instructions for letting go of resentment while practicing forgiveness and gratitude • Instructions for letting go of grudges that muddle mothers' minds and psyches, as well as an emphasis on the role of forgiveness in strengthening positive emotions and turning negative feelings into neutrals, and its role in feelings of calm and adaptability • The role of gratitude in increasing life satisfaction and improving social and marital relationships to reduce negative emotions and stressors associated with the severity of pregnancy nausea and vomiting	completing homework on forgiveness and gratitude
Fifth	Positive social interactions, optimism, and hope	• Improving interpersonal relationships • Training to increase participation in recreational activities • Encouragement to strengthen one's optimism and hope for the future • Instructions for getting rid of pessimistic thoughts and beliefs to improve concentration, reduce stress, and adapt to pregnancy better	the images of an open and a closed door were presented to mothers to help them recall times in their lives when certain doors were closed or open to them
Sixth	A general summary of positive psychology	• Assessing mothers' awareness of positive psychology • Reviewing and summarizing the previous sessions' topics and studying the impact of a positive attitude on pregnancy stress and anxiety	Completing the intervention and filling out the Hamilton questionnaire

After getting to know the client, the counsellor and client's roles, goals, session schedule, and how to complete the questionnaires were all explained in the first session. According to the Ministry of Health of Iran's protocol, the educational content provided to pregnant mothers in the first trimester of pregnancy routinely includes education on pregnancy changes, personal hygiene, and nutrition. In addition to routine training in nausea and vomiting physiology, the present study introduced positive psychology and discussed 24 competencies/capabilities related to positive behaviour. The following sessions were designed to help mothers alleviate pregnancy-related nausea and vomiting symptoms while also strengthening their mental skills. At the end of each session, homework was assigned. The control group only received routine care.

### Outcomes

The current study had a four-week follow-up period, and the study outcome consisted of examining the pattern of coping with pregnancy that the participants completed immediately before the intervention and four weeks after the end of counselling sessions.

#### Data collection tools

The data in this study were collected using the Rhodes nausea and vomiting severity scale, the demographics questionnaire, and the pregnancy adjustment questionnaire.

#### Demographic and midwifery questionnaire

The questionnaire included: age, education, number of previous pregnancies, monthly household income and type of homeownership.

### Coping with pregnancy pattern

Hamilton and Lobe (2008) developed a pregnancy adaptation questionnaire to assess women's coping strategies when facing pregnancy challenges. This questionnaire contains three subscales: 1. preparation and planning (14 questions), 2. avoidance (7 questions), and 3. positive-spiritual coping (5 questions). Cronbach's alpha for the readiness-planning subscale in the early, mid, and late stages of the first pregnancy is 0.82, 0.85, and 0.86, respectively, whereas the avoidance subscale is 0.77 to 0.80 and the positive spiritual subscale is 0.73 to 0.78 in all trimesters [8]. The Persian version of this questionnaire was psychometrically assessed in 2018 by Shobiri et al., and spiritual compatibility and avoidance compatibility was confirmed in the analysis of confirmatory factors with 26 items in three areas of planning and preparation, spiritual compatibility, and avoidance compatibility. Cronbach's alpha calculated the instrument's reliability to be 0.92 [7, 16]. The weighted average of the scores in each domain was calculated to determine the adaptation patterns of women; women with a higher mean in each area tend to cope with stressful situations more effectively, and no cut-off point is specified in this questionnaire.

### Rhode’s nausea and vomiting severity index scale

This questionnaire is intended to assess the amount of nausea, vomiting, and gagging experienced in the previous 12 h. There are eight questions on this tool with 5 Likert scores ranging from 0 to 4. A higher score indicates more severe nausea, vomiting, and gagging. The severity of vomiting nausea is determined by a grading scale; a score of 3–8 signifies mild severity; a score of 9–16 indicates moderate severity; a score of 24–17 indicates severe severity; while a score of 32–34 denotes extreme severity. The validity and reliability of the questionnaire both in the foreign study and in Nurane et al. 's Persian version have been confirmed with a Cronbach's alpha coefficient of 0.89 [17, 18].

### Data analysis and statistic tests

SPSS 16 was used to analyze the information. The Kolmogorov–Smirnov test revealed that the data were normally distributed. We compared qualitative variables by using Chi-square tests and independent t-tests for quantitative variables. The effect of time, group, and interaction between group and time evaluated using repeated measure ANOVA test and MAVOVA test with controlling the number of pregnancies. Effect size < 0.3 indicates a small effect. The level of significance was (*P*** > **0.05).

## Results

### Demographic

The majority of women in the control group were 30–35 years old (43.3%), while the majority of women in the intervention group were 25–30 years old (33.3%) (*p* = 0.323). In terms of the number of pregnancies, the control group was multiple gravida (73.3%), whereas the intervention group was nulligravida (*p* = 46.7%) (*p* = 0.035). In terms of education, the majority of women in the control group had a university education (33.3%), whereas the majority of women in the intervention group had a diploma (26.7%) (*p* = 0.274). In both groups, their husbands had diploma education (*p* = 0.929), the majority of participants were not employed (*p* = 0.912), and their husbands were self-employed (*p* = 0.522). The majority of women in the intervention group had adequate income (30.0%), while the majority of women in the control group had more than adequate income (36.7%) (*p* = 0.188). Personal housing ownership was the highest status in both groups in terms of housing ownership status (*p* = 0.050). In terms of demographic factors, the comparison of the two groups was not statistically significant. However, the difference between the two groups was statistically significant only in terms of the number of pregnancies (Table 2).

**Table 2:** Comparison of the frequency distributions of demographic characteristics in two study groups

Variable		Control group		Intervention group		Chi-square	*P* Value
		Number	%	Number	%		
**Age**	18–20	4	13.3	6	20.0	3.486	0.323
	20–25	8	26.7	5	16.7		
	25–30	5	16.7	10	33.3		
	30–35	13	43.3	9	30.0		
**Gravida**	Primigravida	8	26.7	16	53.3	4.444	0.035
	Multigravida	22	73.3	14	46.7		
**Education**	Primary	5	16.7	4	13.3	5.136	0.274
	Secondary	7	23.3	4	13.3		
	High School	6	20.0	7	23.3		
	Diploma	2	6.7	8	26.7		
	University	10	33.3	7	23.3		
**Job**	No employee	24	80.0	24	80.0	1.200	0.912
	Employee	2	6.7	3	10.0		
	Student	2	6.7	2	6.7		
	Other	2	6.7	1	3.3		
**Family Income**	Less Than Enough	3	10.0	2	6.7	4.783	0.188
	Adequate	9	30.0	9	30.0		
	More than adequate	11	36.7	5	16.7		
	Too Much Is Enough	7	23.3	14	46.7		
**Homeownership**	The Owner	16	53.3	13	43.3	0.5997	0.050
	Rent	7	23.3	15	50.0		
	Relatives	7	23.3	2	6.7		

### Coping with pregnancy pattern

**Fig. 2:**
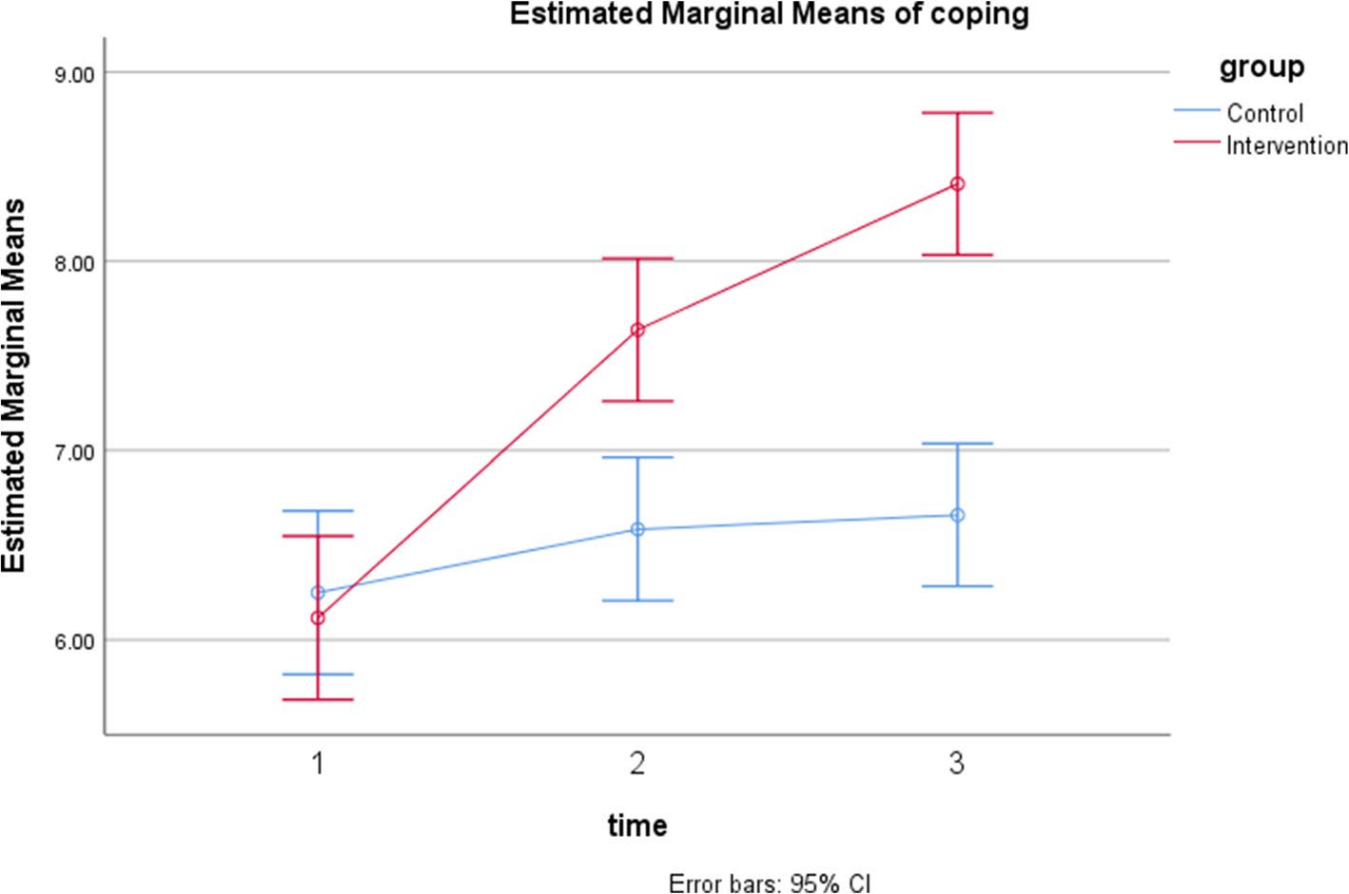
The comparison effect of two groups on coping with pregnancy pattern in three follow up period

In the counseling group, the mean (SD) of the total score of coping with pregnancy before the intervention was 6.11 (1.31) which increased to 8.40 (1.03) in 4 weeks after the intervention. In the control group, it was 6.25 (1.03) before the intervention and increased to 6.66(1.03) in 4 weeks after the intervention. The results of the independent t-test demonstrate that the difference between the two groups in the mean total score of coping was not statistically significant before intervention (*P*-value = 0.974) but after the intervention was significant (*P*-value = 0.001). In addition, based on the MANOVA test with adjusting the number of pregnancies, the mean total score of coping with pregnancy was statistically significant 4 weeks after the intervention compared with the control group (Adjusted mean difference (AMD) = -1.84, CI95% -2.36, -1.32, *p* = 0.001). Also, the mean scores of the preparation and planning pattern (AMD = -1.20, CI95% -1.47, -0.93, *p* = 0.001) and avoidance pattern (AMD = -0.47, CI95% 0.74, -0.21, *p* = 0.001) were statistically significant between the two groups 4 weeks after the intervention but the effect on the positive spiritual pattern was short term (Table 3). The increasing slope of the total score of coping in the control group was milder than in the intervention group (Fig. 2).

**Table 3:** Comparison of coping with pregnancy in the two groups in pre-intervention and at two points of follow-up periods

Coping	Time	Intervention	Control	*P*-value *	AMD **	CI 95%	*P* value ***
		Mean (SD)	Mean (SD)				
**The total score**	Before	6.11(1.31)	6.25(1.03)	0.974	0.01	-0.57, 0.59	0.974
	Immediate	7.63(1.02)	6.58(1.03)	0.001	-1.15	-1.67, -0.64	0.001
	4 weeks	8.40(1.03)	6.66(1.02)	0.001	-1.84	-2.36, -1.32	0.001
**Planning Preparation**	Before	1.54(0.71)	1.48(0.61)	0.581	-0.09	-0.44, 0.24	0.581
	Immediate	2.69(0.38)	1.77(0.57)	0.001	-0.93	-1.19, -0.68	0.001
	4 weeks	2.94(0.36)	1.75(0.62)	0.001	-1.20	-1.47, -0.93	0.001
**Positive spiritual**	Before	2.34(0.68)	2.50(0.53)	0.534	0.09	-0.20, 0.39	0.534
	Immediate	2.71(0.66)	2.54(0.50)	0.001	-0.22	-0.52, 0.06	0.126
	4weeks	2.67(0.59)	2.56(0.49)	0.244	-0.61	-0.11, 0.43	0.244
**Avoidance pattern**	Before	2.23(0.47)	2.26(0.63)	0.943	0.01	-0.28, 0.30	0.943
	Immediate	2.23(0.47)	2.26(0.63)	0.943	0.01	-0.28, 0.30	0.943
	4 weeks	2.79(0.43)	2.34(0.56)	0.001	-0.47	-0.74, -0.21	0.001

*SD* Standard deviation,* Independent T-test, **Adjusted mean difference (AMD), *** Repeated Measure ANOVA was used after the intervention with adjusting gravida

The effect of time, group, and interaction between group and time was evaluated using repeated measure ANOVA. The *P*-value of Mauchly’ test of sphericity is > 0.05 and GG Epsilon is more than 0.75 then we look at Huynh–Feldt results in the 'Tests of Within-Subjects Effects’ table. There was significant interaction between group and time Wilks' Lambada = 0.56, F (2,57) = 22.26, *p* = 0.001, partial eta squared = 0.43. There was a substantial main effect for time with both groups showing a rising in coping score across the three time periods Wilks' Lambada = 0.37, F (2,57) = 47.35, *p* = 0.001, partial eta squared = 0.62. the main effect comparing the two groups was significant Wilks' Lambada = 0.46, F (3,57) = 21.87, *p* = 0.001, partial eta squared = 0.54) and suggest that positive psychology is more effective than routine care.

## Discussion

The present study was aimed to determine the effect of supportive counselling with a positive psychology approach on improving coping patterns in pregnant women who experienced nausea and vomiting. According to our result, supportive counselling accompanied by positive psychology had a positive effect on the total score of coping with pregnancy patterns and two domains (the preparation-planning and avoidance patterns) in the 4-week follow-up but the effect on the positive spiritual pattern was short-term. A similar study was not available but our results are consistent with the findings of the Carissoli et al. study. They investigated the effect of a Positive Psychological Intervention on the adaptation of Italian mothers with pregnancy" in 2019. The results of Carissoli 's study showed that positive orientation improved psychological adjustment in pregnant women [19]. The use of problem-solving coping strategies can significantly reduce the feeling of insecurity in pregnant women and improve their mental health [20, 21]. Schetter showed that pregnant women who were more aware of stress management techniques had more energy and better overall health than other women. He believed that pregnant women who coped well with stress could take better care of themselves [22]. Anxious women are more likely to exhibit the avoidance adjustment pattern. Some of these women may even deny being pregnant, resulting in a miscarriage. In such cases, continued worry and stress lead to weakness, and the person reaches a point where he is unable to cope with the situation and loses motivation. The person will eventually reach a point where mental illness symptoms appear [23, 24].

One of the components of positive psychology is the spiritual and religious dimension, which has been regarded as one of the strategies for women to cope with pregnancy worries [25]. Furthermore, increased satisfaction, self-confidence, and decreased high-risk behaviours suggest that pregnant women benefit from higher levels of spiritual and religious health [26]. Mental health is closely related to adaptation. Lucero (2013) found that reduced enjoyment of spiritual abilities in pregnant women reduces pregnancy satisfaction, increases fear of labor, unpleasant feelings of physical and mental changes during the period, and symptoms of depression and anxiety [27]. Monfared et al. demonstrated that spiritual counselling is an acceptable approach for improving coping patterns among first-time pregnant women in dealing with pregnancy challenges [28]. Study findings suggest that positive psychology can promote positive spiritual and overall adjustment patterns. In addition, the positive effects of counselling with a positive approach in other aspects of health in pregnant women have been reported in some studies such as the one conducted by Mostafaei et al. in 2019, which showed that positive group counselling improved the psychological capital and quality of life of Zanjani pregnant women [14]. Furthermore, in 2017, Rastad et al. showed that positive group counselling increased the happiness of mothers with unwanted pregnancies [29]. The issue of mental health and pregnancy adaptation, as well as its consequences, are essential parts of midwifery care and should be emphasized in prenatal programs to improve women's health and empower them to better control their emotions during pregnancy.

### Strengths of study

All the principles of control trial studies were observed in this study and we don’t have a loss of following in participants. Data collection tools were standard and psychometric properties of the Persian form of the questionnaires have been evaluated based on Iranian culture**.**

### Study limitations

This study was concurrence with the covid-19 pandemic, only volunteers women with mild to moderate NVP attended counselling sessions. Future studies with large sample sizes and longer follow-up periods will be required to draw better conclusions.

## Conclusion

Overall, the findings of this study demonstrated that supportive counselling based on a positive approach was effective in improving coping patterns in pregnant women who experienced nausea and vomiting. Furthermore, the effectiveness of the intervention continued to four weeks after the end of the sessions. To maximize the benefits of this approach, training packages addressing positive content should be developed and used in prenatal care.

## Supplementary Information

**Additional file 1. **

## Data Availability

The dataset used in the present study is available from the corresponding author upon reasonable request.

## Abbreviations

NVP: Pregnancy nausea and vomiting
AMD: Adjusted mean difference
SD: Standard Deviation
CI: Confidence Interval
MANOVA: Multivariate Analysis of Variance
