# Supplementary material for: Effects of supportive counseling using a positive psychology approach on coping patterns among pregnant women with nausea and vomiting
Source: BMC Pregnancy Childbirth. 2022 Mar 27;22:259. doi: 10.1186/s12884-022-04603-4 (PMC8958341; doi:10.1186/s12884-022-04603-4)

**Study aim IRCT**

The Effect of Counseling Using Positive Approach on Maternal Adaptation and Severity of Pregnancy Nausea and Vomiting

**Design**

The Present Study Is Phase 3 Of A Randomized Clinical Trial, Has A Control Group With Parallel Groups. That Will Be Performed On 60 Patients (30 Interventions And 30 Control Interventions). We Do Not Have Blinding. Eligible Women Will Be Selected By Convenience Sampling Method And Will Be Assigned To Two Intervention And Control Groups Using 4 Random Blocks.

**Settings and conduct**

The Study Population Includes Women with Nausea and Vomiting That Will be Selected Using the convenience Sampling Method and then Randomly Assigned to the Intervention and Control Groups by the 4 Block Design.

**Participants/Inclusion and exclusion criteria**

Inclusion Criteria: Having 3-16 Scores Based on the Reduz Pregnancy Nausea and Vomiting Questionnaire, Gestational Age 6-10 Weeks, Age 18-35 Years, Wanted Pregnancy, Normal Pregnancy and Access to Telephone. Exclusion criteria: Having High-Risk Pregnancy Symptoms, Stressful Events, Unwillingness to Continue Participating in The Study.

**Intervention groups**

The Intervention Group Six Sessions of Individual Positive Counseling Based on The Silgman Protocol (2006) Will be Done With Emphasizing the three Paths to Happiness (Enjoyment, Commitment, and Meaning in Life). Each Session Will be Done 45 Minutes in 3 Times a Week. The Control Group will Receive Routine Care.

**Main outcome variables**

Coping and Severity of Nausea and Vomiting in Pregnancy

General information

**Reason for update**

Changing the start time of sampling

**Acronym**

**IRCT registration information**

IRCT registration number: **IRCT20150731023423N17**

Registration date: **2020-10-31, 1399/08/10**

Registration timing: **prospective**

Last update: **2020-11-02, 1399/08/12**

Update count: **1**

**Registration date**

2020-10-31, 1399/08/10


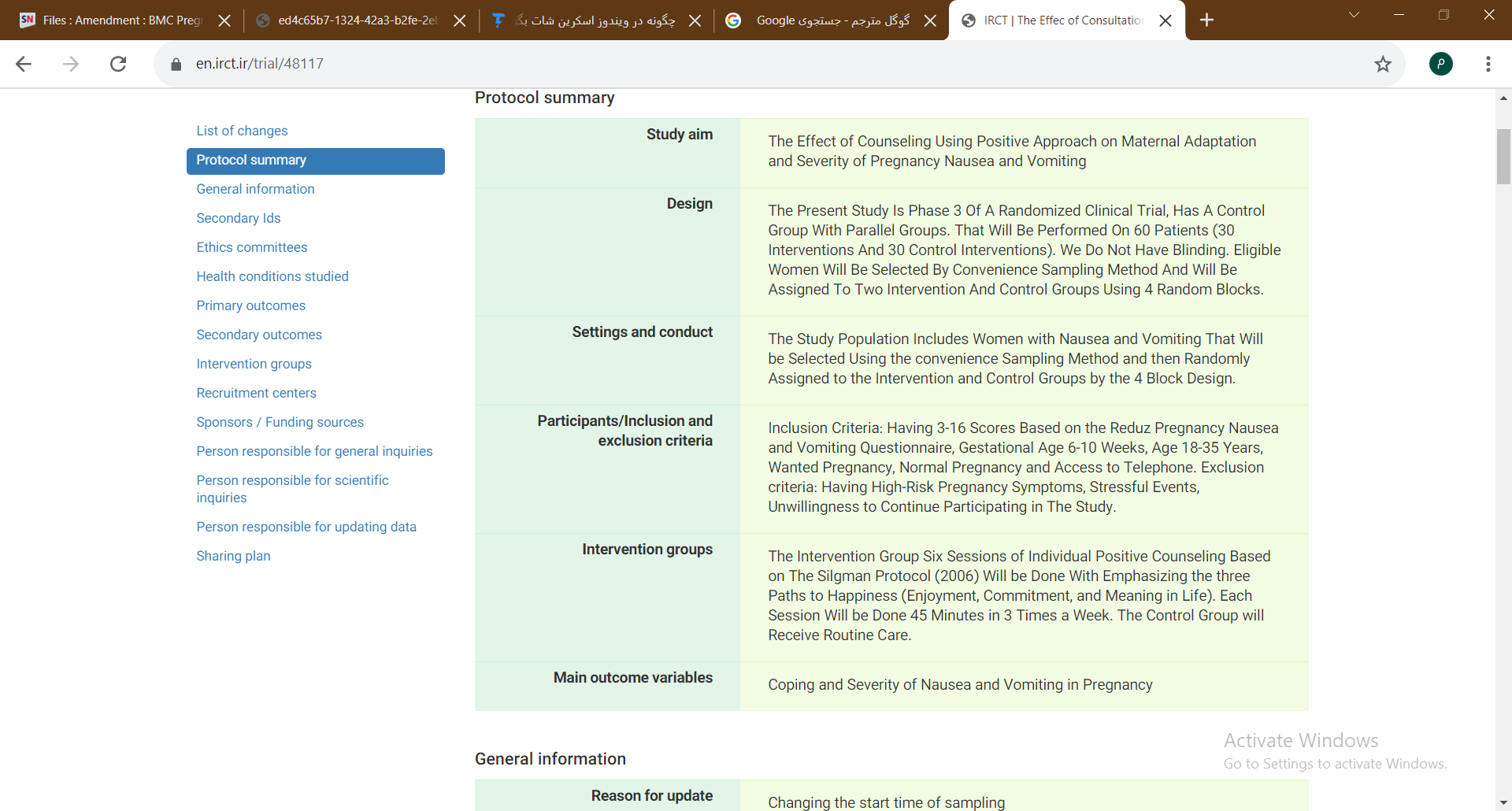


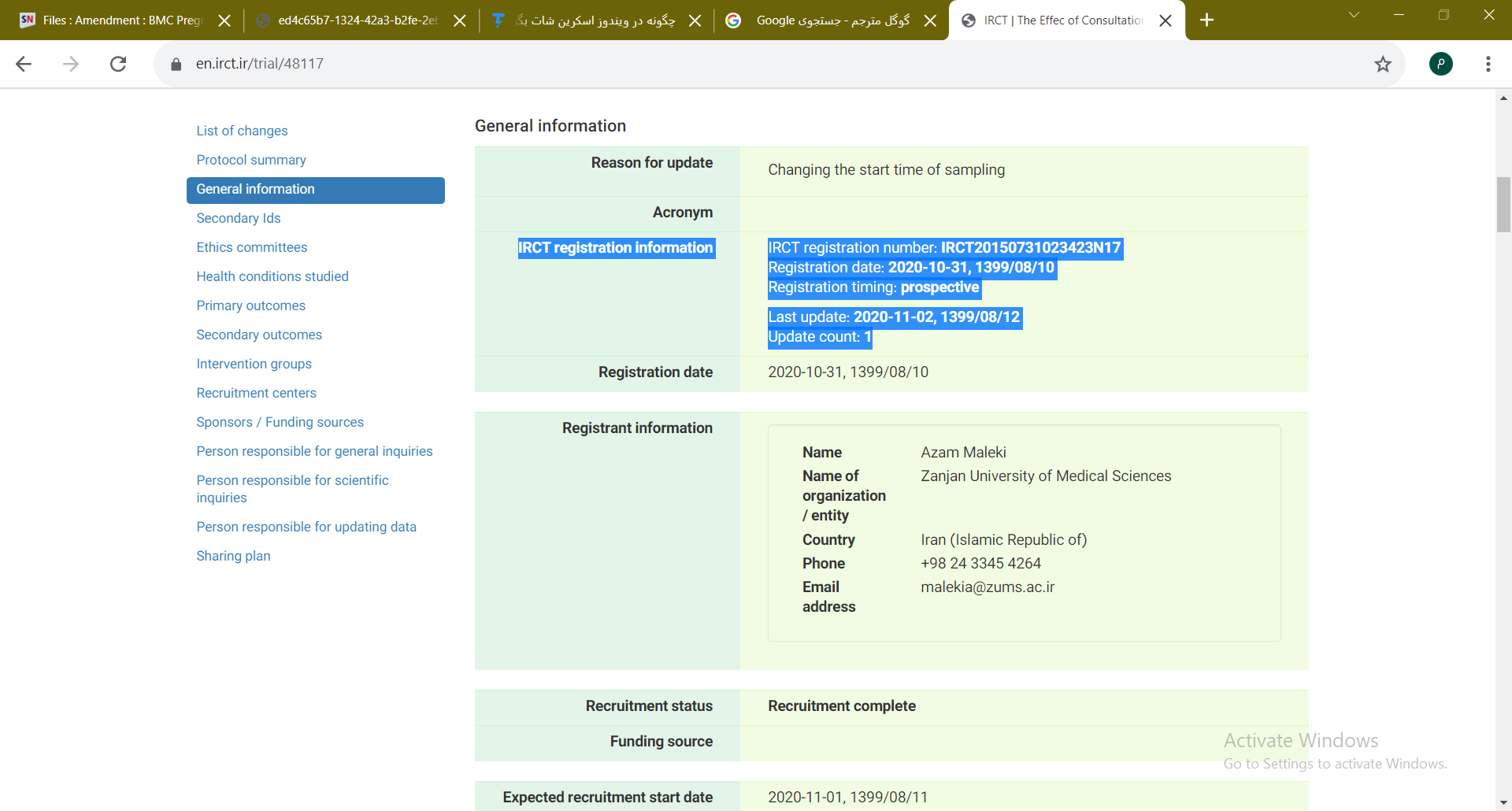

Supplement: Supplementary file 1 — Additional file 1. [file 12884_2022_4603_MOESM1_ESM.docx]
